# Myocardial cathepsin D is downregulated in sudden cardiac death

**DOI:** 10.1371/journal.pone.0230375

**Published:** 2020-03-16

**Authors:** Yu Kakimoto, Ayumi Sasaki, Maki Niioka, Noboru Kawabe, Motoki Osawa

**Affiliations:** 1 Department of Forensic Medicine, Tokai University School of Medicine, Isehara, Kanagawa, Japan; 2 Support Center for Medical Research and Education, Tokai University, Tokyo, Kanagawa, Japan; Indiana University, UNITED STATES

## Abstract

Cathepsins are the major lysosomal proteases that maintain intracellular homeostasis. Herein, we investigated the alterations in myocardial cathepsin expression during aging, cardiac hypertrophy, and sudden cardiac death (SCD). Cardiac tissue and blood were sampled from autopsy cases. Subjects were classified into three groups: SCD with cardiac hypertrophy (SCH), compensated cardiac hypertrophy (CCH), and control. Immunoblotting was performed for the major cardiac cathepsins and their targets: cathepsin B, D, and L (CTSB/D/L), p62, ATP synthase subunit c (ATPSC), and α-synuclein (ASNC). Immunohistochemical analysis and ELISA using serum samples were performed for CTSD. Cardiac CTSB and CTSD were upregulated with age (r = 0.63 and 0.60, respectively), whereas the levels of CTSL, p62, ATPSC, and ASNC remained unchanged. In age-matched groups, cardiac CTSD was significantly downregulated in SCH (*p* = 0.006) and CTSL was moderately downregulated in CCH (*p* = 0.021); however, p62, ATPSC, and ASNC were not upregulated in cardiac hypertrophy. Immunohistochemistry also revealed decreased myocardial CTSD levels in SCH, and serum CTSD levels were relatively lower in SCH cases. Overall, these results suggest that upregulation of cardiac CTSB and CTSD with age may compensate for the elevated proteolytic demand, and that downregulation of CTSD is potentially linked to SCH.

## Introduction

The lysosome is a single-membrane cytoplasmic organelle with an acidic pH of approximately 4.0–5.0 and contains over 50 hydrolases [1]. Lysosomal proteolysis is essential for maintaining intracellular homeostasis, and cathepsins (CTSs) are the primary lysosomal proteases. CTSs are categorized into three types on the basis of the amino acid residues in their active site: serine CTSs (CTSA/G), cysteine CTSs (CTSB/C/F/H/K/L/O/S/V/W/X), and aspartic CTSs (CTSD/E) [2]. Alterations in CTS expression have been reported primarily in neurodegenerative diseases: CTSB and CTSD are upregulated in the brain during aging [3], Parkinson’s disease [4], and Alzheimer’s disease [5,6]. In contrast, cardiac CTS levels in heart failure (HF) display phase-dependent changes; CTSD is upregulated in the subacute phase after myocardial infarction and is downregulated in the chronic phase [7,8] and in the final stage of dilated cardiomyopathy [9]. Serum CTS levels are reportedly useful predictors of the prognosis of myocardial infarction [10–12]. However, alterations in cardiac CTS levels during aging and sudden cardiac death (SCD) are yet unclear.

SCD is defined as an unexpected death without any obvious noncardiac cause, occurring within 1 h of symptom onset (witnessed), or within 24 h of being in normal health (unwitnessed) [13–15]. Despite the overall decline in cardiovascular mortality over the last decade, SCD is a persistent issue [16–18], and recent data suggest that the incidence of SCD is approximately 70 to 100 per 100,000 individuals per year in developed countries [17,19]. As SCD is the major cause of natural sudden death, its antemortem prevention and postmortem diagnosis are socially important. Most SCD victims do not have heart disease or some have heart disease without severe systolic dysfunction [20,21]. However, retrospective studies including autopsies have reported that numerous SCD cases have coronary atherosclerosis and left ventricular hypertrophy [22,23]. Therefore, it is generally accepted that the primary direct cause of SCD is malignant ventricular arrhythmias based on the structural and electrical remodeling secondary to coronary artery disease and pressure overload [24,25]. Studies are required to elucidate myocardial molecular changes that contribute to pathological remodeling. Here, we hypothesized that impaired lysosomal proteolytic function contributes to pathological remodeling prior to SCD. We investigated the alterations in myocardial CTS expression during aging, cardiac hypertrophy, and SCD. In particular, we focused on CTSB, CTSD, and CTSL, which are abundantly expressed in the human heart, and on their target proteins, namely p62, which is the major autophagic substrate and accumulates under lysosomal dysfunction [26], ATP synthase subunit c (ATPSC), which accumulates upon CTSD impairment [27], and α-synuclein (ASNC), which is degraded by CTSB and CTSL [28, 29].

## Materials and methods

### Subjects

SCD was defined as unexpected death without obvious noncardiac causes occurring within 24 h of being in normal health [14,22]. Because cardiac hypertrophy is an established risk factor for SCD [25, 30], subjects were placed into three groups according to the cardiac size, namely SCD with cardiac hypertrophy (SCH), compensated cardiac hypertrophy (CCH), and control. Cardiac hypertrophy was defined as hypertrophy with a ratio of heart weight to body height >2.5 g/cm [31]. SCH cases consisted of ischemic HF, hypertensive HF, and aortic stenosis. None of the patients in this study had hypertrophic cardiomyopathy. CCH and control cases did not exhibit severe coronary atherosclerosis or HF symptoms and died of noncardiac causes. The clinical characteristics of the subjects are summarized in Table 1 and S1 Table. All the bodies were moved to a 4°C refrigerator within 24 h of death, and autopsy was performed within a week.

**Table 1:** Characteristics of the cases.

	SCH (n = 11)	CCH (n = 10)	Cont (n = 17)
**Cause of Death**	Ischemic HF (6), Hypertensive HF (4), Aortic stenosis (1)	Accident (9), Noncardiac disease (1)	Accident (14), Noncardiac disease (3)
**Age**	62.0 ± 16.0 (41–85)	58.5 ± 18.0 (31–86)	50.2 ± 20.9 (17–88)
**Sex (m/f)**	9/2	8/2	12/5
**Postmortem interval (days)**	2.4 ± 1.5	3.0 ± 0.9	3.4 ± 1.8
**BMI (kg/m**^**2**^**)**	27.1 ± 8.2*	24.7 ± 2.9*	20.6 ± 2.8
**Heart weight**	553 ± 202**	486 ± 43**	314 ± 64
**Coronary atherosclerosis**	Severe (Ischemic HF) None–Little (Others)	None–Mild	None–Mild

SCH, sudden cardiac death with cardiac hypertrophy; CCH, compensated cardiac hypertrophy; HF, heart failure; BMI, body mass index; Coronary atherosclerosis is categorized according to the most serious occlusion at cross section: severe > 90%, high > 75%, mild > 50%, little > 25%, none ≤ 25%.

* *p* < 0.05

** *p* < 0.001, compared with Cont. Individual characteristics are detailed in S1 Table.

### Sampling

Approximately 1-cm-thick transverse sections of the heart (located 2–3 cm under the atrioventricular sulcus) were sampled at autopsy. About 1 cm^3^ myocardia was dissected from the middle layer of the left lateral ventricular wall, which does not include trabecula or epicardial adipose tissue, and immediately immersed in liquid nitrogen and stored at -80°C until protein isolation. Other cardiac tissues were fixed in 10% formalin for histological examination. Subsequently, samples for microscopy were obtained from the left ventricular anterior, lateral, and posterior walls, the septum, and the right ventricular wall. Histological examination was performed by a skilled pathologist in a blinded manner. Blood samples were obtained from the left ventricular chamber. This study was approved by the Ethics Committee of Tokai University and informed consent was obtained from the bereaved relatives of the subjects. The study protocol conformed to the ethical guidelines of the 1975 Declaration of Helsinki.

### Western blotting

Approximately 100 μg of frozen cardiac sample was homogenized in 500 μL of RIPA buffer (Wako, Japan), and the supernatant was harvested after centrifugation at 15,000 rpm for 10 min. Protein concentration was determined using the BCA Protein Assay (Thermo Fisher Scientific, USA). Western blotting was performed for CTSB, CTSD, CTSL, p62, and ASNC using capillary immunoblotting with the Wes system (Protein Simple, Japan) in accordance with the manufacture’s protocol. Briefly, 5 μL of 1 μg/μL protein sample was applied to a 12–230 kDa Wes Separation Module, and probed with the first antibody for 1 h, which was followed by probing with the secondary antibody for 30 min. Western blotting for ATPSC was performed manually: 10 μL of a 0.02 μg/μL protein sample was applied to a 4%–12% Bis-Tris Plus gel (Invitrogen Waltham, USA), and electrophoresis was carried out in NuPAGE^™^ MES SDS Running Buffer (Thermo Fisher Scientific) for 18 min at 200 V. The electrophoresed proteins were transferred to the membrane using Trans-Blot Turbo Transfer System (Bio-Rad) at 25 V and 2.5 A for 1 h. The membrane was probed with the primary antibody overnight at 10°C, and then with the secondary antibody for 1 h at room temperature. After application of Immobilon Western HRP Substrate (Merck, USA), signal intensity was measured with a CCD camera. The antibodies used in this study and their dilutions were as follows: anti-CTSB at 1:500 (AF953, Novus Biologicals, USA), anti-CTSD at 1:50 (AF1014, Novus Biologicals), anti-CTSL at 1:200 (AF952, Novus Biologicals), anti-p62 at 1:50 (GP62-C, Progen, Germany), anti-ATPS at 1:5000 (ab181243, Abcam, UK), and anti-ANSC at 1:100 (10842–1, Proteintech, Japan). Anti-GAPDH (G9545, Sigma, Japan) was used for the internal control at a dilution of 1:500 for capillary immunoblotting and 1:5000 for manual immunoblotting. Anti-rabbit HRP-linked antibody (NA9340, GE Healthcare Life Sciences, Japan) was used at a dilution of 1:5000 for manual immunoblotting.

### Immunohistochemistry

The transverse section of the heart was stained with hematoxylin and eosin and examined under a microscope. For immunostaining of CSTD, antigen retrieval was carried out by heating in Target Retrieval Solution, pH 9.0 (Dako, USA) at 120°C for 10 min. Blocking was carried out with Protein Block, Serum-Free (Dako) at room temperature for 10 min. The specimens were immunostained with anti-CTSD antibody (1:100; Novus Biologicals) at 4°C overnight, and then with the secondary antibody at room temperature for 1 h. DAB and hematoxylin dye were used for staining.

### Serum CTSD assay

The level of CTSD in the serum was quantified using the Cathepsin D Human ELISA kit (Abcam) in accordance with the manufacture’s protocol. The subjects who had brain damage were excluded because CTSD is highly expressed in the brain. The serum CTSD levels were compared between age-matched groups.

### Statistical analysis

Quantitative data were imported into Excel 2010 (Microsoft, USA) and presented as means ± standard deviation values. The correlation between the CTS level and age was assessed by determining the correlation coefficient (r). A regression analysis was performed, and the coefficient of determination (R^2^) was calculated according to the least squares method. Multiple comparisons were made using the Steel–Dwass test in Excel Statistics 2015 (SSRI, Tokyo, Japan). Differences with *p* values <0.05 were considered statistically significant.

## Results

### Expression levels of cardiac CTSs and their substrates during aging

**Fig 1:**
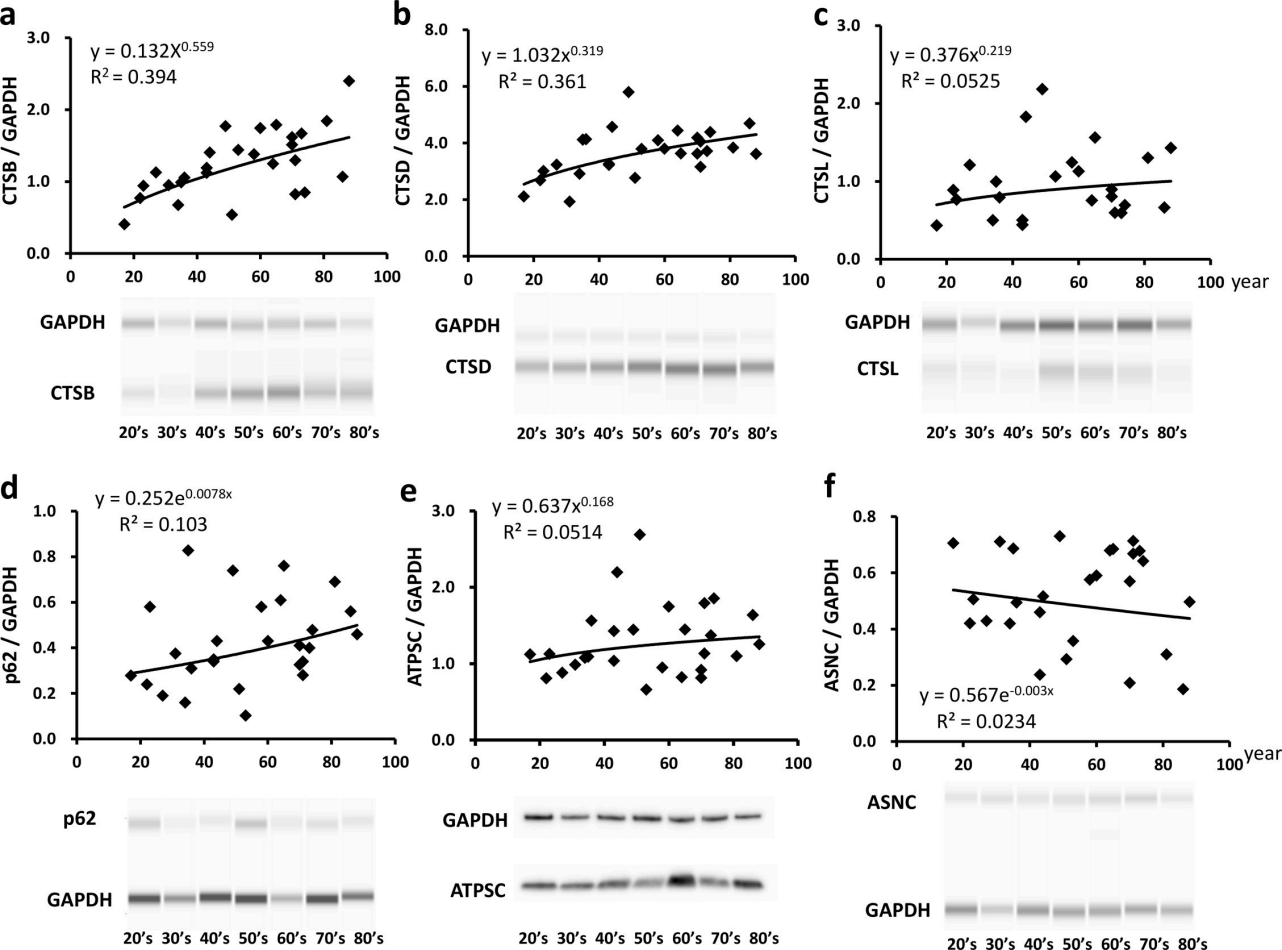
Alterations in the expression levels of cardiac cathepsins and their substrates with age. Relative expression levels of cardiac cathepsin B (CTSB, a), cathepsin D (CTSD, b), cathepsin L (CTSL, c), p62 (d), ATP synthase subunit c (ATPSC, e), and α-synuclein (ASNC, f) in cases of noncardiac death are shown with representative images of immunoblots. n = 27 in each graph. The uncropped images of the immunoblots are presented in S1–S6 Figs.

In subjects with noncardiac cause of death, cardiac CTSB and CTSD were upregulated and displayed a moderate correlation with aging (r = 0.628 and 0.601 respectively, Fig 1A and 1B); however, cardiac CTSL levels were not significantly correlated with aging (r = 0.229, Fig 1C). Among the substrate proteins, the levels of p62, one of the primary substrates in lysosomal autophagy [26], were slightly, but not significantly, increased (r = 0.321, Fig 1D). Furthermore, ATPSC, the primary substrate of CTSD [27], and ANSC, a common substrate for CTSB and CTSL [29], were not upregulated upon cardiac aging (r = 0.227 and 0.153 respectively, Fig 1E and 1F). Thus, none of these CTS substrates were observed to accumulate in the heart with age. The original uncropped images of the immunoblots are presented in S1–S6 Figs.

### Expression levels of cardiac CTSs and their substrates in SCD

**Fig 2:**
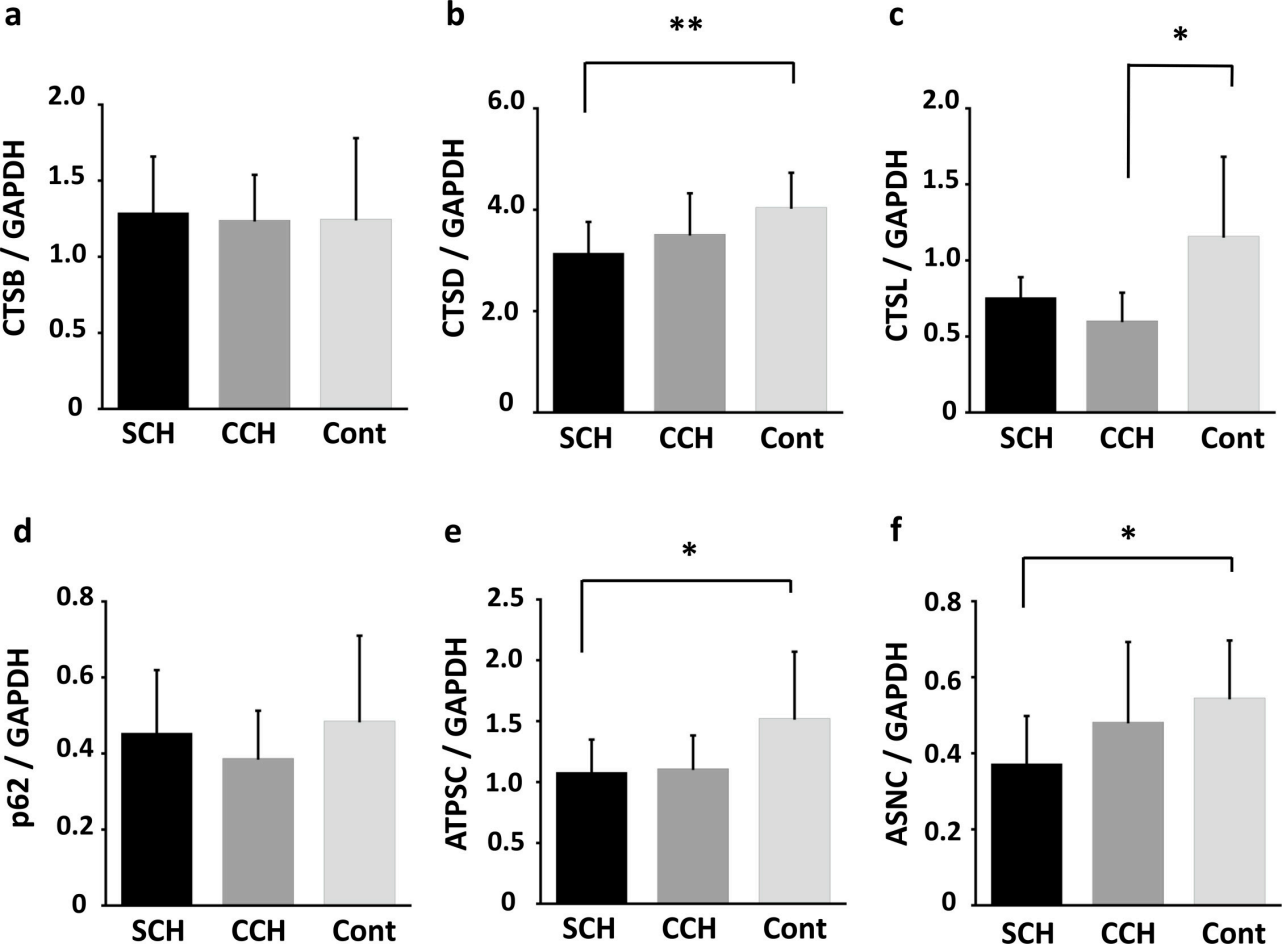
Alterations in the expression levels of cardiac cathepsins and their substrates in sudden cardiac death. Relative expression levels of cardiac cathepsin B (CTSB, a), cathepsin D (CTSD, b), cathepsin L (CTSL, c), p62 (d), ATP synthase subunit c (ATPSC, e), and α-synuclein (ASNC, f) in sudden cardiac death with cardiac hypertrophy (SCH, n = 11), compensated cardiac hypertrophy (CCH, n = 10), and control (n = 13) groups. All groups are age-matched. * *p* < 0.05; ** *p* < 0.01. The uncropped images of the immunoblots are presented in S1–S6 Figs.

Among the age-matched groups, cardiac CTSB levels remained largely unchanged (Fig 2A); however, cardiac CTSD was significantly downregulated in SCH (*p* = 0.006) and slightly downregulated in CCH (Fig 2B). Moreover, CTSL was moderately downregulated in CCH (Fig 2C, *p* = 0.021). Among the CTS substrates, p62 levels remained unchanged in cardiac hypertrophy (Fig 2D), whereas cardiac ATPSC and ASNC were moderately downregulated in SCH (Fig 2E and 2F, *p* = 0.048 and *p* = 0.039, respectively). The uncropped images of the immunoblots are presented in S1–S6 Figs.

### Cardiac histopathology with CTSD

**Fig 3:**
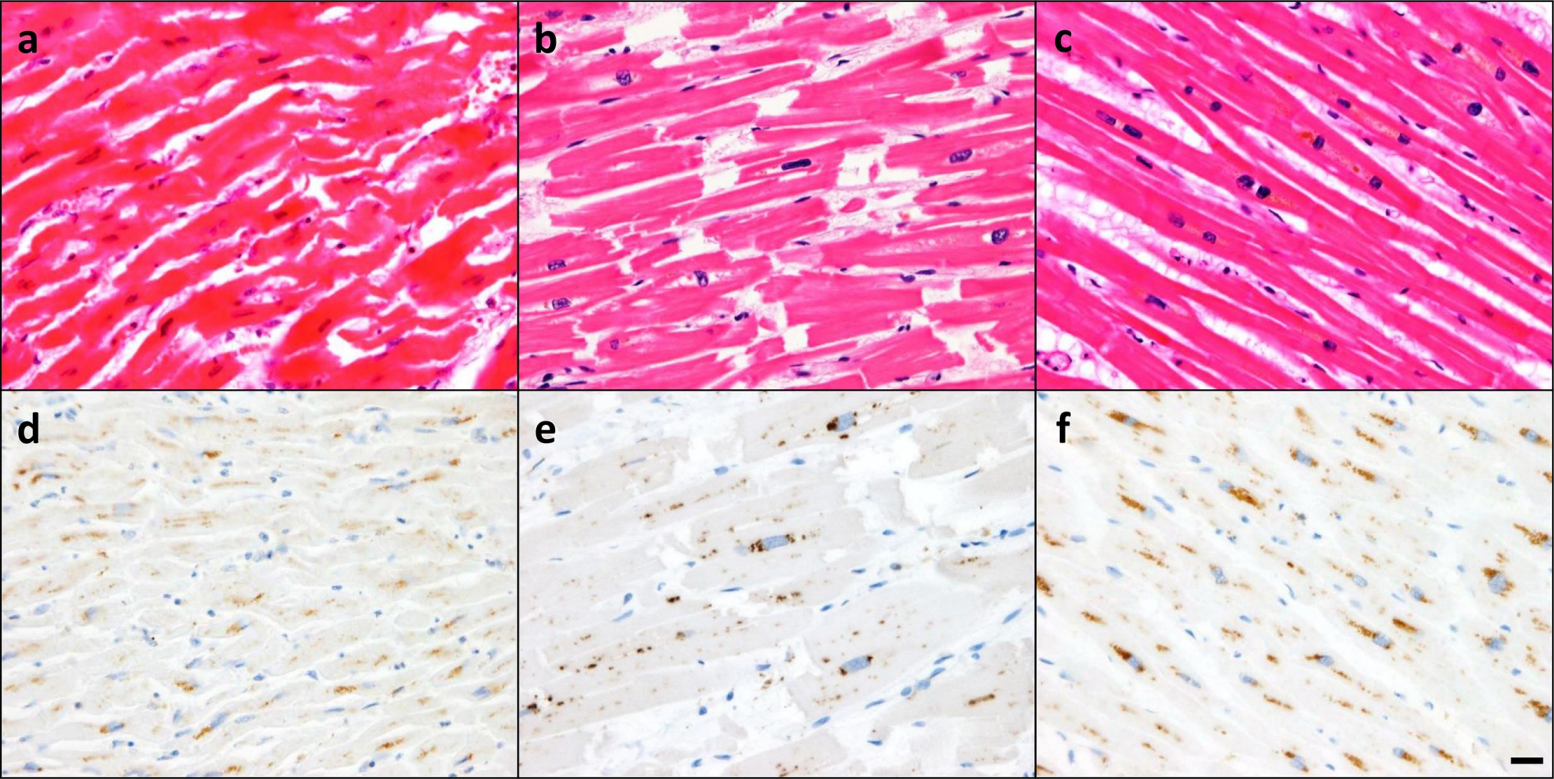
Expression pattern of cardiac cathepsin D. Representative microscopic images; (a-c) hematoxylin and eosin staining (d-f) cathepsin D (CTSD) immunostaining. Expression levels (a, d) at 45 years in sudden cardiac death with cardiac hypertrophy (SCH) patient with ischemic heart failure, (b, e) at 31 years in compensated cardiac hypertrophy (CCH) subject with accidental death, (c, f) at 71 years in the control case with accidental death. Bar = 20 μm.

In the cases of SCD with ischemic HF, cardiomyocytes showed eosinophilic waviness, contraction band necrosis, and slight neutrophil infiltration, which represent the acute phase of myocardial infarction (Fig 3A). Moreover, cardiomyocyte hypertrophy with nuclear enlargement was equal in SCH and CCH, and was accompanied by a slight increase in interstitial fibrosis (Fig 3B). The hearts of control individuals displayed a normal cardiomyocyte array; however, some elderly subjects showed moderate interstitial fibrosis (Fig 3C).

CTSD accumulation was observed as a cytoplasmic granular deposit in cardiomyocytes, specifically around the perinuclear area (Fig 3D–3F). Although this distribution pattern was independent of cardiac pathology, CTSD density was decreased in SCH and increased in older individuals. No marked CTSD accumulation was observed in vascular endothelial cells or fibroblasts.

### Alterations in serum CTSD levels

**Fig 4:**
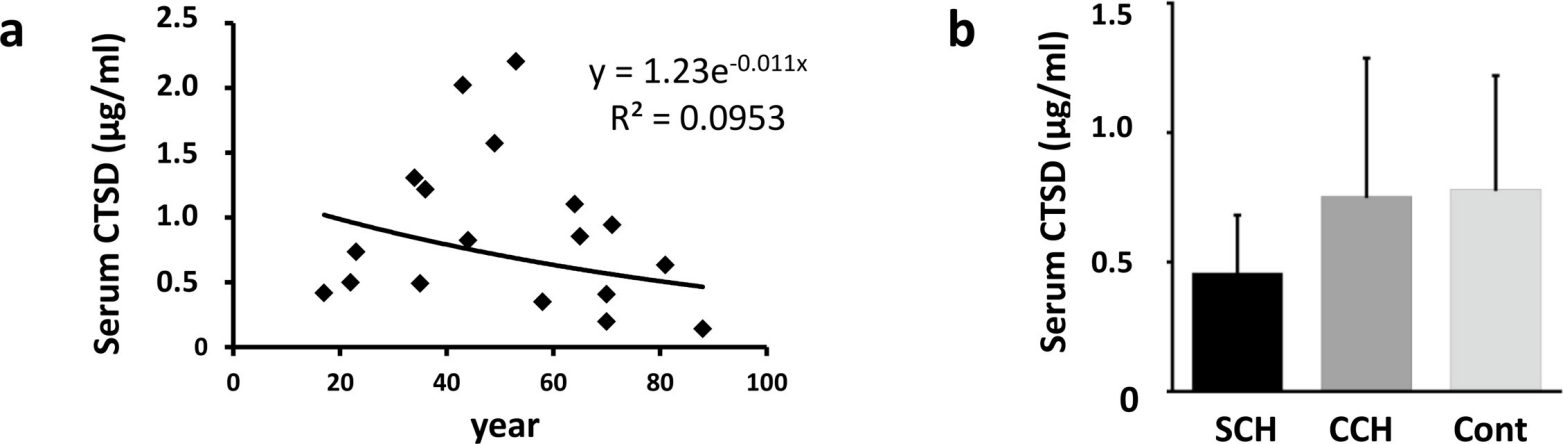
Serum cathepsin D levels. (a) Serum CTSD levels at different ages. (b) Comparison of serum CTSD levels among SCH, CCH, and control groups.

No significant changes were observed in serum CTSD levels with age (r = 0.309, Fig 4A). Also, no significant changes were observed in serum CTSD levels in SCH, CCH, and control subjects (Fig 4B).

## Discussion

### Changes in the expression of cardiac CTSs with aging

The present results show that cardiac CTSB and CTSD were stably upregulated with age, whereas CTSL levels remained unchanged. Heterogeneous alterations in the levels of CTSs indicate that the expression of lysosomal CTSs is regulated differently with cardiac aging.

Numerous studies have reported that brain CTSD activity is increased up to 2-fold in aged rats compared to that in young adults [3, 32, 33] and that CTSD protein level is increased up to 10-fold in the rat brain [34, 35]. The discrepancy in the levels and enzymatic activity of CTSD indicates the accumulation of inactive CTSD with aging. Therefore, our results probably show the levels of both the active and inactive forms of CTSD. Similarly, in the human brain, CTSD levels and activity are reported to increase up to 2-fold with age; therefore, the enzymatic activity mostly correlates with the CTSD expression levels [6,36]. No changes in the levels of CTSD substrates observed in the present study indicate that the lysosomal proteolytic activity is generally maintained in the aged human heart. This is in agreement with previous reports, wherein it has been shown that autophagosome clearance is preserved in human cardiac aging [37,38]. Thus, the age-dependent upregulation of cardiac CTSD might be able to cater to the proteolytic demand during aging.

### Cardiac CTSs in cardiac hypertrophy and SCD

In this study, CTSB and CTSL were found to be downregulated in cardiac hypertrophy, potentially implying slightly limited myocardial proteolytic activity. However, the levels of their substrate proteins, including ATPSC and ASNC, remained unchanged or were relatively downregulated. This suggests that the downregulation of CTS can be balanced, to some extent, by the downregulation of redundant proteins during hypertrophic growth of cardiomyocytes. Cardiac hypertrophic remodeling may be divided into two types based on clinical findings: physiological remodeling in CCH and pathological remodeling in SCH. Cardiac immunoblotting results obtained in the present study indicate that advanced downregulation of CTSD is potentially associated with pathological remodeling.

Oxidative stress results in cytosolic release of lysosomal CTSD [39], and some clinical studies have reported that circulating CTSD levels are elevated after myocardial infarction until 6 months later [11,40]. Hence, we analyzed serum CTSD levels to determine whether myocardial CTSD is downregulated in SCH by assessing the CTSD leakage into the blood. Consequently, serum CTSD levels in SCH were observed to be relatively lower in our study, and we assume that myocardial CTSD leakage is not the primary cause of myocardial CTSD reduction in SCH. These results are in agreement with those of previous studies showing that low serum CTSD levels predict an unfavorable prognosis after myocardial infarction, including in-hospital mortality and cardiac dysfunction [10,11].

CTSD degrades various substrate proteins. Thus, alterations in CTSD expression levels and activity are potential physiological adaptations or lethal pathologies depending on the situation. Because CTSD has high sequence homology with renin, it potentially displays renin-like activity [41,42]. Therefore, the elevation of circulating CTSD after myocardial infarction potentially contributes to angiotensin formation and an increase in blood pressure [40]. However, CTSD yields a cleaved 16-kDa form of prolactin, which has antiangiogenic and proapoptotic properties, and contributes to postpartum cardiomyopathy [43]. In contrast, CTSD-deficient (CTSD^-/-^) mice develop normally for 2 weeks after birth but die with intestinal necrosis, thromboembolism, and lymphopenia within 4 weeks [44]. Although the heart of CTSD^-/-^ mice shows restrictive cardiomyopathy along with myocardial ATPSC and LC3-II deposits, its ejection fraction is maintained [8, 27, 45]. Moreover, CTSD heterozygous mice (CTSD^+/-^), which do not present with cardiac dysfunction under normal conditions, display impaired autophagic flux and exacerbated cardiac function after myocardial infarction [8].

Overall, this study shows that CTSD downregulation in the human heart can potentially contribute to not only HF but also SCD through myocardial and electrical remodeling. However, as we analyzed autoptic samples with various postmortem interval, some postmortem degradation may have confounded the immunoblotting results. With such a practical limitation, our results do not indicate the target protein of CTSD crucial for lethal arrhythmogenesis. Therefore, further studies are required to focus on cardiac hypertrophy and SCD, to decipher the precise role of myocardial CTSD in SCH.

## Supporting information

S1 Table Characteristics of individual subjects. (PDF)

S1 Fig Capillary immunoblotting of cardiac CTSB and GAPDH. Exposure was set automatically with the Wes system. Con13, Con8, CCH4, Con6, Con4, CCH3, and CCH7 were as represented in Fig 1A.
(PDF)

S2 Fig Capillary immunoblotting of cardiac CTSD and GAPDH. Exposure was set automatically with the Wes system. Con13, Con8, CCH4, Con6, Con4, CCH3, and CCH7 were as represented in Fig 1B.
(PDF)

S3 Fig Capillary immunoblotting of cardiac CTSL and GAPDH. Exposure was set automatically with the Wes system. Con13, Con8, CCH4, Con6, Con4, CCH3, and CCH7 were as represented in Fig 1C.
(PDF)

S4 Fig Capillary immunoblotting of cardiac p62 and GAPDH. Exposure was set automatically with the Wes system. Con13, Con8, CCH4, Con6, Con4, CCH3, and CCH7 were as represented in Fig 1D.
(PDF)

S5 Fig Immunoblotting of cardiac ATPSC and GAPDH. The membrane was cut at around 25 kDa and independently stained with the primary antibody. Exposure time was set at 30 s for ATPSC detection, and 10 s for GAPDH detection. Dashed lines indicate cropped immunoblots presented in Fig 1E.
(PDF)

S6 Fig Capillary immunoblotting of cardiac ASNC and GAPDH. Exposure was set automatically with the Wes system. Con13, Con8, CCH4, Con6, Con4, CCH3, and CCH7 were as represented in Fig 1F.
(PDF)
