# Supplementary material for: Myocardial cathepsin D is downregulated in sudden cardiac death
Source: PLoS One. 2020 Mar 16;15(3):e0230375. doi: 10.1371/journal.pone.0230375 (PMC7075574; doi:10.1371/journal.pone.0230375)
Supplement: S1 Table — (PDF) [file pone.0230375.s001.pdf]

S1Table.

Characteristics of individual subjects.

ID	Cause of death	Age(y)	Sex	Postmortem interval (day)	BMI (kg/m2)	Heart weight (g)	Coronary atherosclerosis
SCH1	Hypertensive HF	74	M	2.2	26.0	407	None
SCH2	Hypertensive HF	80	M	2.0	19.3	494	None
SCH3	Ischemic HF	65	F	1.4	21.1	404	Severe
SCH4	Ischemic HF	45	F	2.0	20.0	477	Severe
SCH5	Ischemic HF	54	M	0.8	23.9	560	Severe
SCH6	Ischemic HF	48	M	2.6	23.8	408	Severe
SCH7	Ischemic HF	41	M	1.7	27.9	425	Severe
SCH8	Ischemic HF	85	M	2.1	26.2	450	Severe
SCH9	Hypertensive HF	66	M	6.8	34.5	596	Little
SCH10	Hypertensive HF	42	M	1.6	49.8	1104	Little
SCH11	Aortic stenosis	82	M	3.4	25.6	758	Little
CCH1	Accident	71	M	1.9	26.6	550	Mild
CCH2	Accident	64	M	2.0	26.1	413	Little
CCH3	Accident	70	M	1.8	22.4	448	Mild
CCH4	Accident	43	M	3.5	28.4	497	None
CCH5	Accident	73	M	2.9	24.0	545	Little
CCH6	Accident	70	F	2.6	20.0	481	Little
CCH7	Accident	86	F	2.5	20.6	516	Little
CCH8	Accident	34	M	4.5	28.1	456	Little
CCH9	Putamen hemorrhage	43	M	3.8	23.5	467	None
CCH10	Accident	31	M	4.0	27.2	450	Little
Con1	Accident	44	M	0.9	22.3	374	None
Con2	Pneumonia	65	F	1.2	14.7	218	None
Con3	Alcohol cirrhosis	49	M	1.2	18.9	270	None
Con4	Accident	60	M	4.5	21.9	361	Little
Con5	Accident	58	M	2.1	23.8	331	None
Con6	Accident	53	M	3.5	25.3	383	None
Con7	Accident	36	M	4.4	20.6	354	None
Con8	Accident	35	M	3.2	25.4	339	None
Con9	Accident	23	M	5.0	17.5	306	None
Con10	Accident	27	M	1.8	24.3	369	None
Con11	Aortic dissection	81	M	3.3	20.2	380	Mild
Con12	Accident	88	F	2.2	18.1	259	Little
Con13	Accident	22	M	6.7	19.7	247	None
Con14	Accident	17	F	1.8	18.5	166	None
Con15	Accident	74	F	6.4	20.1	254	Little
Con16	Accident	71	F	4.5	18.5	352	Little
Con17	Accident	51	M	5.4	20.7	366	None
