# Supplementary material for: Myocardial cathepsin D is downregulated in sudden cardiac death
Source: PLoS One. 2020 Mar 16;15(3):e0230375. doi: 10.1371/journal.pone.0230375 (PMC7075574; doi:10.1371/journal.pone.0230375)

S1 Fig.

Capillary immunoblotting of cardiac CTSB and GAPDH. Exposure was set automatically with the Wes system. Con13, Con8, CCH4, Con6, Con4, CCH3, and CCH7 were as represented in Fig 1a.

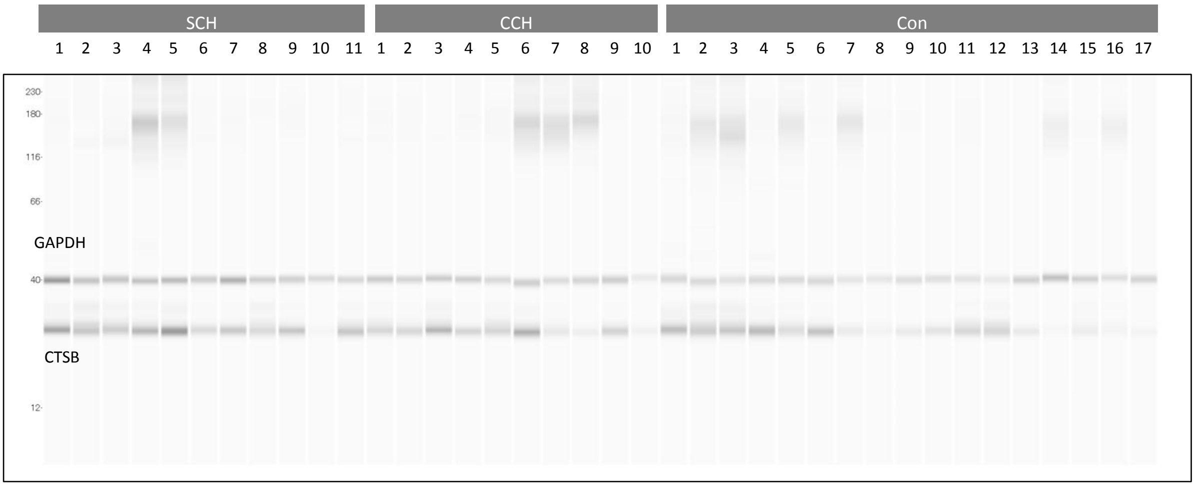

Supplement: S1 Fig — Exposure was set automatically with the Wes system. Con13, Con8, CCH4, Con6, Con4, CCH3, and CCH7 were as represented in Fig 1A. (PDF) [file pone.0230375.s002.pdf]
