# Supplementary material for: Myocardial cathepsin D is downregulated in sudden cardiac death
Source: PLoS One. 2020 Mar 16;15(3):e0230375. doi: 10.1371/journal.pone.0230375 (PMC7075574; doi:10.1371/journal.pone.0230375)
Supplement: S5 Fig — The membrane was cut at around 25 kDa and independently stained with the primary antibody. Exposure time was set at 30 s for ATPSC detection, and 10 s for GAPDH detection. Dashed lines indicate cropped immunoblots presented in Fig 1E. (PDF) [file pone.0230375.s006.pdf]

S5 Fig.

Immunoblotting of cardiac ATPSC and GAPDH. A membrane was cut at around 25 kDa and independently stained with the primary antibody. Exposure time was set at 30 s for ATPSC detection, and 10s for GAPDH detection. Dashed lines indicate cropped immunoblots presented in Fig 1e.

WB1

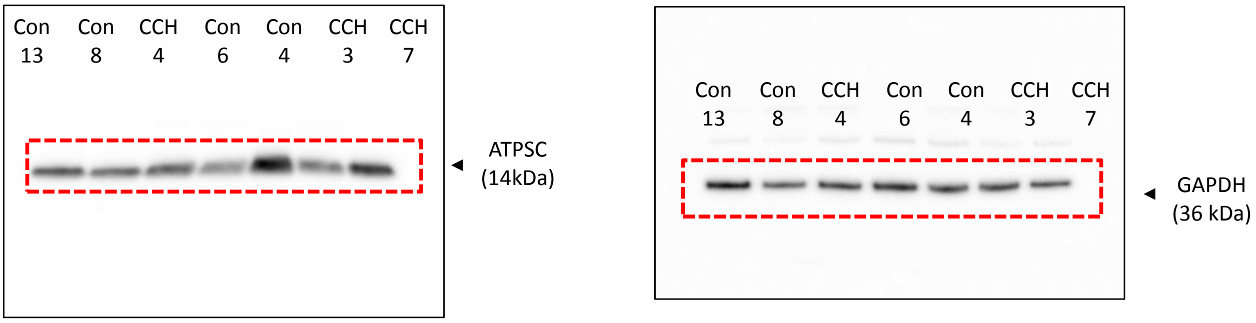

WB2

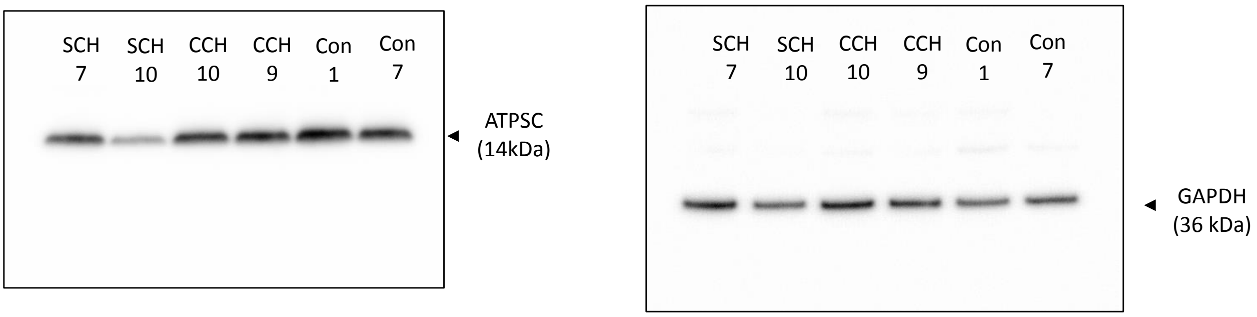

WB3

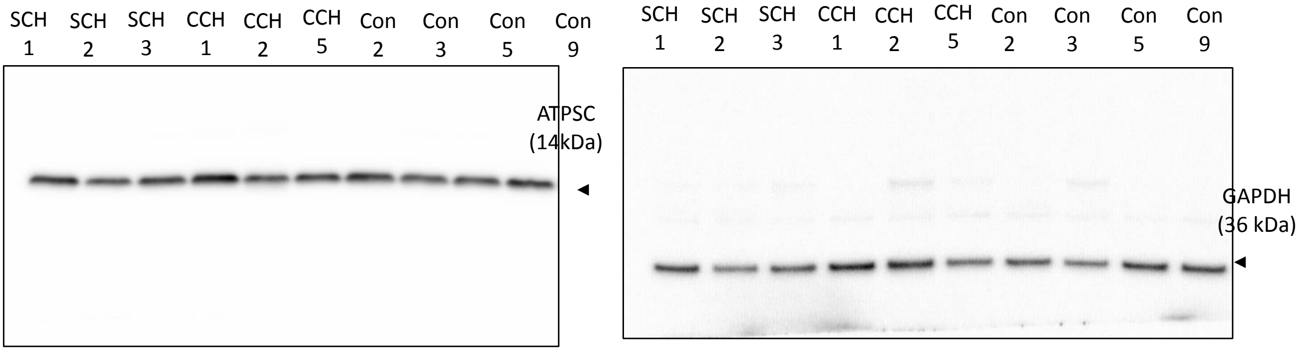

WB4

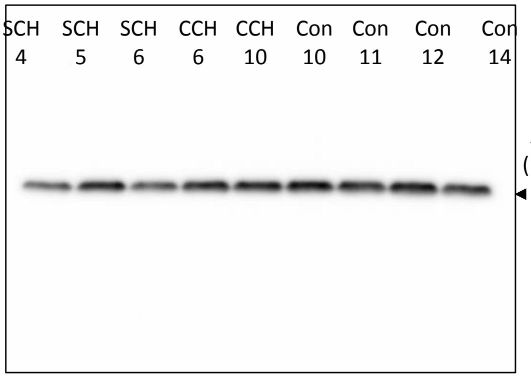

ATPSC (14kDa)

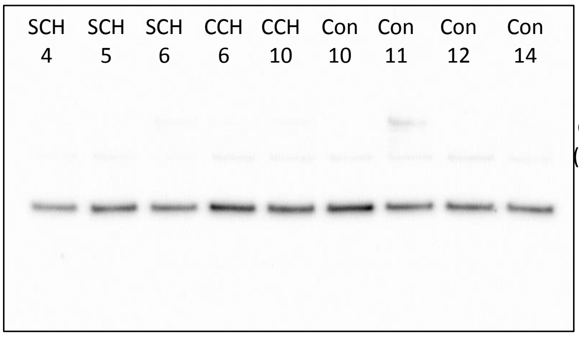

GAPDH (36 kDa)

WB5

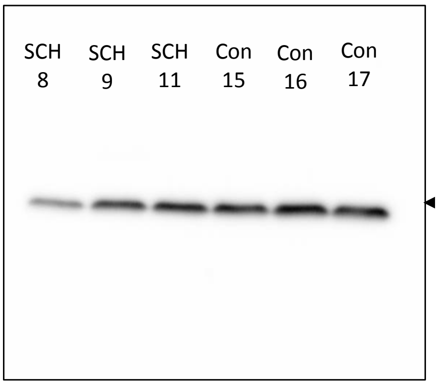

ATPSC (14kDa)

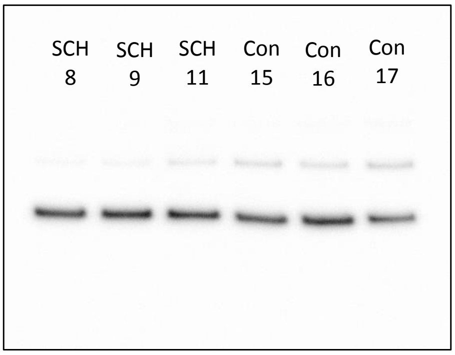

GAPDH (36 kDa)
